# Hydraulic endorectal actuator for prostate radiotherapy reduces variations in motion in a silicone rectal phantom

**DOI:** 10.3389/fonc.2026.1686529

**Published:** 2026-02-11

**Authors:** Aryan Niknam Maleki, Mark Runciman, Julia Murray, George Mylonas

**Affiliations:** 1 The Hamlyn Centre, Department of Surgery and Cancer, Imperial College London, London, United Kingdom; 2 Department of Radiotherapy and Imaging, The Institute of Cancer Research and The Royal Marsden NHS Foundation Trust, London, United Kingdom

**Keywords:** hydraulic actuator, prostate cancer, prostate radiotherapy, rectal motion, rectal phantom, rectal stabilizer

## Abstract

**Background:**

The accuracy and morbidity of prostate cancer radiotherapy are influenced by unpredictable variations in rectal filling and patient motion. We developed a soft robotic hydraulic endorectal actuator that aims to reduce rectal motion and retract the rectum to restore the anorectal angle, improve target accuracy, and reduce toxicity during prostate cancer radiotherapy. The ability of the endorectal actuator to stabilize the rectum and improve prostate radiotherapy outcomes has not yet been assessed. This study evaluates the actuator’s performance in a simulated rectal phantom.

**Methods:**

We fabricated a rectal phantom using silicone and motor-controlled elastic ribbons to simulate muscle tone and control the phantom diameter. The rectal compliance of the phantom was validated using a barostat balloon and was deliberately set low to simulate a high resistance to distension to challenge the actuator’s capabilities. We assessed the actuator’s ability to (1) resist dynamic peristaltic forces and (2) reproduce the rectal position and anorectal angle from varying initial displacements. The anterior–posterior rectal diameter and anterior rectal wall (ARW) displacements were measured using video tracker software.

**Results:**

The phantom demonstrated a rectal compliance of 4.19 ml/mmHg within the 40 ml–60 ml volume range, meeting the low-compliance target. During dynamic compression, the endorectal actuator reduced the change in the anterior–posterior diameter and ARW displacement from 25 mm and 15 mm, respectively, to less than 5 mm in both. The actuator reduced the increase in rectal volume from 132.3 cm^3^ (control) to 59.7 cm^3^ (actuator). When the phantom was translated anteriorly, the actuator reduced the anorectal angle deviation from +12° to +2° and anterior displacement of the ARW from 13 mm to 4 mm.

**Conclusion:**

Within this rectal phantom, the endorectal actuator reduced the variations in rectal motion. These findings suggest that the actuator may improve target accuracy and reduce radiation-induced toxicity in prostate radiotherapy, pending *in vivo* validation of the results.

## 1 Introduction

Radiotherapy is a common treatment for prostate cancer and is used to target the prostate bed in a post-prostatectomy setting. Modern hypofractionation techniques and additive pelvic radiotherapy have improved biochemical recurrence rates, with the rate being approximately 5% (1) in radical prostate radiotherapy. While the rate is higher in the post-prostatectomy setting (7%–10%), relapse commonly occurs outside the treatment field, at pelvic nodes, or at metastatic sites (2, 3). However, gastrointestinal toxicity remains a prevalent issue. Due to the proximity of the anterior rectal wall (ARW) to the target volume, this area receives a substantial radiation dose, leading to acute grade ≥2 gastrointestinal toxicity in 16%–38% of prostate radiotherapy patients (4–6) and up to 30% of post-prostatectomy patients (7), and late grade ≥2 GI toxicity in 1.5%–3% and 3%–4.5% of patients, respectively (5–8). Variations in rectal motion, caused by peristaltic forces and gas (9), lead to inter- and intra-fraction prostate motion errors and rectal exposure to radiation. The existing management of rectal and prostate motion in prostate radiotherapy includes tracking methods and rectal-stabilizing devices. Tracking methods include online adaptive radiotherapy and CyberKnife^®^, which can improve the accuracy of the delivery beam; however, these methods can be time-consuming and costly (10–13). Rectal stabilizers, such as endorectal balloons (ERBs) and ProSpare™, aim to stabilize the rectum and minimize variations in rectal volume. While ERBs are well tolerated because of their inflatable nature (14), their impact on inter-fraction motion is inconclusive (15–24), and their lack of self-insertion by the patient can lead to errors in the depth of insertion and worsening dosimetry (25, 26). ProSpare™ is a rigid obturator that reduces motion and improves anorectal dosimetry (27–29). It is self-insertable and contains radiopaque steel ball bearings for image guidance. However, its rigidity, which aids in stabilizing the rectum, affects some patients’ ability to self-insert (30).

We previously presented a soft hydraulic endorectal actuator designed to be soft on insertion and rigid upon inflation to stabilize the rectum (31). Initial validations used benchtop force/torque sensors but lacked clinically applicable simulations. In this study, we fabricated and validated of a rectal phantom with biomechanical properties—compliance and capacity—representative of a healthy human rectum. The phantom uses motor-controlled elastic ribbons that simulate peristaltic variations in rectal motion and tone, allowing us to assess the device’s ability to resist dynamic compression and reduce variations in rectal volume. We also aimed to assess the device’s ability to reproduce rectal volume and anorectal angle upon insertion. Reproducing the anorectal angle would allow the device to stabilize the rectum and act as a spacer by pulling the ARW away from the target volume. These assessments gauge whether the device improves radiotherapy outcomes.

## 2 Materials and methods

The properties that the phantom must emulate for mechanical representation are rectal anatomy, accurate fracture and tone properties, and the ability to translate and rotate about the anorectal angle. The anatomical properties of the rectum to emulate are rectal diameter and wall thickness, anal canal length, rectal length, and anorectal angle. **Table 1** summarizes the quantitative ranges for emulating these properties with references.

**Table 1:** Desired ranges of anorectal properties to emulate in the rectal phantom.

Property	Range	References
Rectal diameter	30 mm–40 mm	(32, 33)
Wall thickness	1.6 mm–4.5 mm (median 2.6 mm)	(34)
Anal canal length	25 mm–53 mm	(35, 36)
Rectum length	80 mm	(33)
Anorectal angle	85°–125°	(37)
Tensile strength	0.84 MPa	(38)
Rectal compliance	Low	(39–41)

Dal Lago et al. reported the radius of the rectum at first sensation or sensation of gas at low pressured distension using a barostat balloon and MRI. They reported a rectal radius of 19.9 mm using MRI and 18.7 mm using an electronic barostat at a pressure of 7 mmHg. The lowest rectal cross-sectional area reported by Dall et al. (32) was approximately 800 mm^2^ at a distension pressure of less than 2 mmHg. This corresponds to a radius of approximately 16 mm. To reflect this range, the rectal phantom was designed as a silicone cylinder capable of contracting to a diameter of 30 mm–40 mm, which represents the rectum at rest. Rectal wall thickness, ranging from 1.6 to 4.5 mm (34), affects fracture properties and is important for safety assessment of the device. Initial attempts to cast a phantom with a 1.5 mm wall failed because of wall tearing during silicone demolding. A 2.5 mm rectal wall was used instead. The anal canal ranged from 25 mm to 53 mm in length. Following Dal Lago et al. (33), who assumed an 8-cm long cylinder in their volume and radius calculations, a straight 80 mm cylinder was chosen for the phantom. This excludes the sacral flexure (42), which is not a region of interest for the clinical assessment of the endorectal actuator. The anorectal angle ranges from 85° to 125° (37). Here, a thick elastic band that wraps around the phantom is incorporated, inspired by the defecatory model of Stokes et al. (38).

Emulating mechanical properties was emphasized to directly assess the radial stiffness of the endorectal actuator in a more clinical context. Cadaveric studies have shown that the ultimate tensile strength of passive rectal tissue is 0.84 MPa, with failure at 62% strain (43). Ecoflex™ 00–10 silicone, with a tensile strength of 0.827 MPa (44), was selected to match this. Matching the tensile strength ensured that the phantom exhibited realistic fracture properties. However, this silicone has a low elastic modulus (0.05 MPa) (45), which does not adequately represent the rectal tone. *In vivo*, the rectum is an active organ that resists expansion (32, 40, 41), maintaining itself within a physiological expansion range of 0%–35% (38). Rectal compliance values vary widely within the literature: Papaconstantinou reported a normal range of 3 ml/mmH–15 ml/mmHg (39); Van den Berg et al. found a mean rectal compliance of 16 ml/mm with a 5th–95th percentile range of 12 ml/mmHg–20 ml/mmHg in their study of healthy adolescents (41); and Fox et al. reported a mean rectal compliance of 11.9 ml/mmHg (range 13 ml/mmHg–38 ml/mmHg) (40). Additionally, the volume ranges corresponding to these compliance ranges are unclear. Because of the large reported variations in rectal compliance, a phantom with deliberately low compliance was designed. This approach enabled a more rigorous assessment of the endorectal actuator. If the endorectal actuator can reproducibly expand the phantom to a stable volume despite the phantom’s high resistance to distension, the actuator is likely to perform reliably in patients and improve the radiotherapy outcomes.

### 2.1 Fabrication of phantom

**Figure 1:**
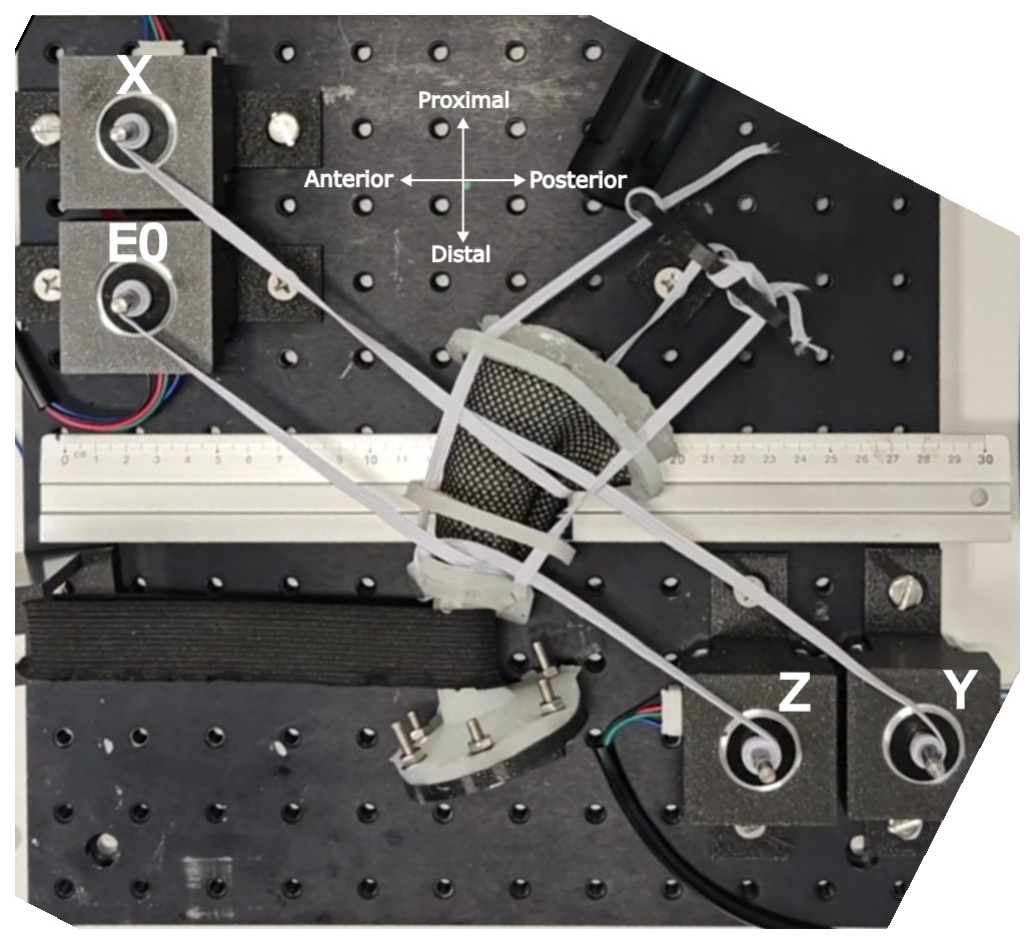
Rectal phantom used to validate the endorectal actuator. Stepper motors are labeled. The orientation cross shows the anatomical direction of the phantom.

The rectal phantom was fabricated by casting silicone (Ecoflex™ 00-10) in a 3D-printed cylindrical mold. The cure formed a 120 mm long tapered cylinder with an inner diameter of 50 mm at the proximal (rectal) end and 23 mm at the distal (anal) end. The “anal canal” is 25 mm in length and conically tapers to a 50-mm rectal diameter. A tapered design was chosen because the rectum narrows as it transitions to the anal canal. The phantom had a wall thickness of 2.5 mm. A silicone flange was attached to the distal end and bolted to a 3D printed flange to suspend the phantom in air. Additional flanges were placed at 30 mm, 70 mm, and 120 mm along the length, allowing the elastic bands to connect the phantom to the proximal flange. This proximal flange suspended the phantom in air while permitting anterior-posterior motion. Tone was induced using four NEMA17 stepper motors and elastic ribbons (Trimming Shop, Hamburg, Germany). To reduce the friction between the ribbons and silicone material, a fabric mesh was wrapped around the silicone rectum before wrapping the ribbons around it. Two elastic ribbons (5 mm wide and 1 mm thick) were wrapped around the motor shafts before being looped twice around the phantom. The proximal and distal ribbons were 60 mm and 50 mm in length, respectively. A thick elastic band (50 mm wide and 5 mm thick) was placed 25 mm from the distal end and looped around a 3D printed hook that was bolted in place. This band simulates the puborectalis muscle and creates an angle in the rectum. **Figure 1** shows a beam-eye view of the phantom with labeled directional references and motors. A 30 cm ruler was placed on the optical platform for image and video calibration.

### 2.2 Validation of phantom

A barostat balloon was used to assess whether the rectal phantom was mechanically representative of a healthy rectum. The phantom was validated using a barostat balloon connected to a rapid barostat bag pump (Mui Scientific, model #P1-RBB-1). The barostat balloon was inserted into the rectal phantom with a water-based lubricant until the 5 cm mark aligned with the phantom’s “anal canal.” This is in line with the manufacturer’s instructions (46). On the pump, the maximum bag pressure was set to 40 mmHg, maximum bag volume to 700 ml, inflation rate to 120 ml/min, and deflation rate to 180 ml/min. A rapid barostat bag (RBB) test was performed by following the instructions displayed on the pump screen: the balloon was emptied and then automatically inflated while recording the pressure and volume. The balloon was inflated to the maximum bag pressure. No sensory tests were performed. Raw data from each RBB test were exported via USB and analyzed using MS Excel. Rectal capacity tests were performed across four levels of simulated “muscular tone,” determined by the number of revolutions each elastic ribbon made around the motor shaft. The first level of tone was defined as barely taut elastic ribbons around the phantoms. The number of revolutions of the elastic ribbons around each motor shaft was noted for this level of tone. The tone was increased by one additional revolution around each motor shaft, up to three additional revolutions. Rectal compliance was taken as the slope of the pressure-volume curves over three volume ranges: 40 ml–60 ml, 60 ml–100 ml, and 100 ml to rectal capacity. These ranges were chosen because a steep inflation curve below 40 ml was observed, where the balloon filled an empty rectum with no external resistance. Rectal compliance should decrease with increasing volume (32), and this property was also validated in the phantom. The 40 ml–60 ml range is most relevant because this is the endorectal actuator’s operating range and the deployed volume of the inflated actuator.

### 2.3 Validating the elastic ribbons

The elastic ribbons used to contract the rectum were validated to ensure consistent stress-strain behavior. The consistent contraction of the elastic ribbons ensured that the motor-controlled elastic ribbons provided reproducible volume changes in the rectal phantom. Reproducible volume changes are required to draw reliable conclusions regarding the clinical efficacy of the endorectal actuator. The elastic ribbons were laid flat on a desk, with one end fixed with duct tape. The other end had a small slit to hook a spring gauge. A 5 m tape ruler was placed next to the ribbon to measure the changing length of the ribbon throughout the experiment. The spring gauge was used to extend the elastic ribbon, and the new lengths of the ribbon were measured with every 0.2 N increase on the spring gauge until either the ribbon was pulled loose from the duct tape or the spring gauge reached a force of 3 N. The strains were calculated by dividing the displacements of the elastic ribbon by its initial length. Starting lengths of 20 cm, 40 cm, 60 cm, 70 cm, and 80 cm were used. Each starting length underwent three trials of the same ribbon and two additional trials with a “new” ribbon of the same length that had not yet been stretched. A “new” ribbon to a “used” ribbon to test whether the elastic ribbons underwent plastic deformation.

**Figure 2:**
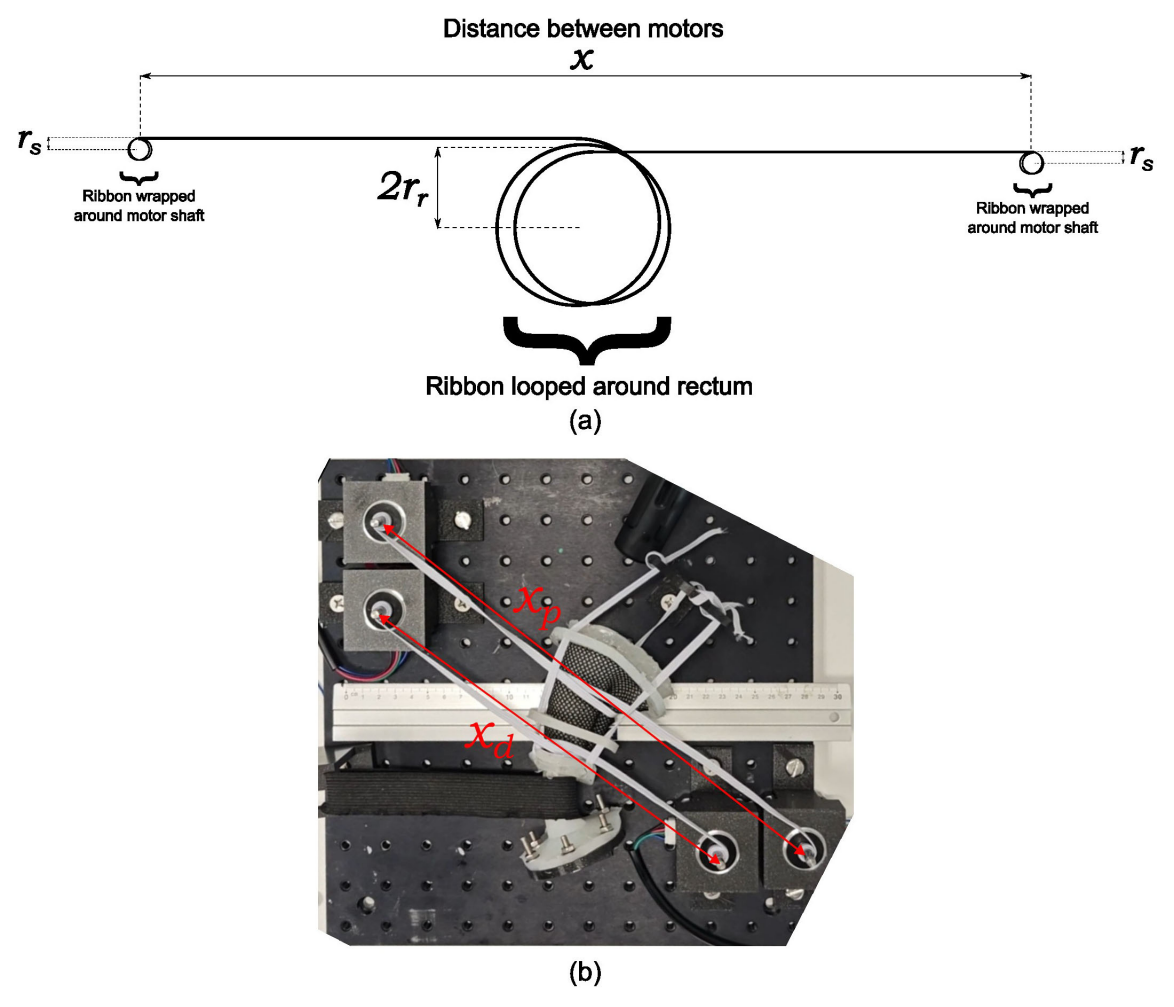
Dimensions of the rectal phantom used to indirectly determine the total length of the elastic ribbons. **(A)** Labeled sketch of the transverse view of rectal phantom. **(B)** Overhead view of the phantom with motor-to-motor distances labeled.

At each tone level in the rectal phantom, the outer diameters at the distal and proximal ribbon sites were measured using a digital caliper. Using these measurements, the strain experienced by the elastic ribbons at each tone level was calculated. because direct measurements of the ribbon length during the contraction of the rectal phantom were not possible, the ribbon length was estimated indirectly based on the dimensions of the phantom. **Figure 2A** shows a transverse view of the elastic ribbons and their dimensions in the rectal phantom. This is related to the phantom setup shown in **Figure 1**, but is shown again in **Figure 2B**. The total length of the elastic ribbons is the sum of the distance between the motor shafts, the circumference around the rectum multiplied by the number of loops the ribbon makes around the rectum, and the length around each motor shaft. The last term, the length around each motor shaft, is challenging to determine because it is related to the number of revolutions the ribbon makes around the shaft. Because the thickness of the ribbon is small relative to the radius of the shaft, each additional revolution significantly increases the radius covered by the next revolution. The distance the ribbon covers around the first revolution around the motor shaft is given by the circumference of the motor shaft. As the elastic ribbon stacks on itself with each revolution, the radius of the circle it covers increases by the thickness of the ribbon for each additional revolution. This increases the circumference covered. The circumference covered by the ribbon in the nth revolution is expressed as Equation 1:

(1) $$C\left(n\right)=2\pi \left(r_{s}+t_{e}\left(n-1\right)\right)$$

where r_s_ is the radius of the motor shafts and t_e_ is the thickness of the elastic ribbon. The length of the ribbon around one motor shaft after n revolutions is therefore the sum of the series (Equation 2).

(2) $$\sum_{i=1}^{n}2\pi \left(r_{s}+t_{e}\left(n-1\right)\right)=t_{e}n^{2}\pi +n\pi \left(2r_{s}-t_{e}\right)$$

Therefore, the total length of the elastic ribbon, $L_{e}$, around the rectal phantom is expressed in Equation 3 as:

(3) $$L_{e}=x+2l\pi r_{r}+2\left(t_{e}n^{2}\pi +n\pi \left(2r_{s}-t_{e}\right)\right)$$

Because some of these terms are constants specific to the phantom, the equation can be simplified. To the nearest cm, the distance between the proximal motor shafts, X and Y, was measured to be 33 cm, and the distance between the distal shafts, Z and E0, was 25 cm. The elastic ribbon only looped around the phantom once, and the thickness of the elastic ribbon was 1 mm. The radius of the shaft was 2.5 mm. Therefore, the simplified equations expressing the lengths of the proximal and distal elastic ribbons in Equations 4 and 5, respectively, are in mm:

(4) $$L_{e,\;proximal}=330+5\pi +2\left(n^{2}\pi +4n\pi \right)$$

(5) $$L_{e,distal}=250+5\pi +2\left(n^{2}\pi +4n\pi \right)$$

Using Equations 4 and 5, the length of the elastic ribbons was calculated at each tone level, which increased with each additional revolution. The length of the ribbon at each tone level was subtracted from the original length of the ribbon to obtain the strain, δ_e_, expressed as a percentage in Equation 6:

(6) $$\epsilon_{e}=\frac{L_{e}-L_{\text{initial}}}{L_{e}}\times 100\%$$

where L_initial_ is the original length of the elastic ribbon of the sensor. The strain was calculated for both the proximal and distal elastic ribbons. This is expected to differ between the proximal and distal ribbons, as the rectal radii and distance between the motor shafts at these positions are different.

### 2.4 Device fabrication

**Figure 3:**
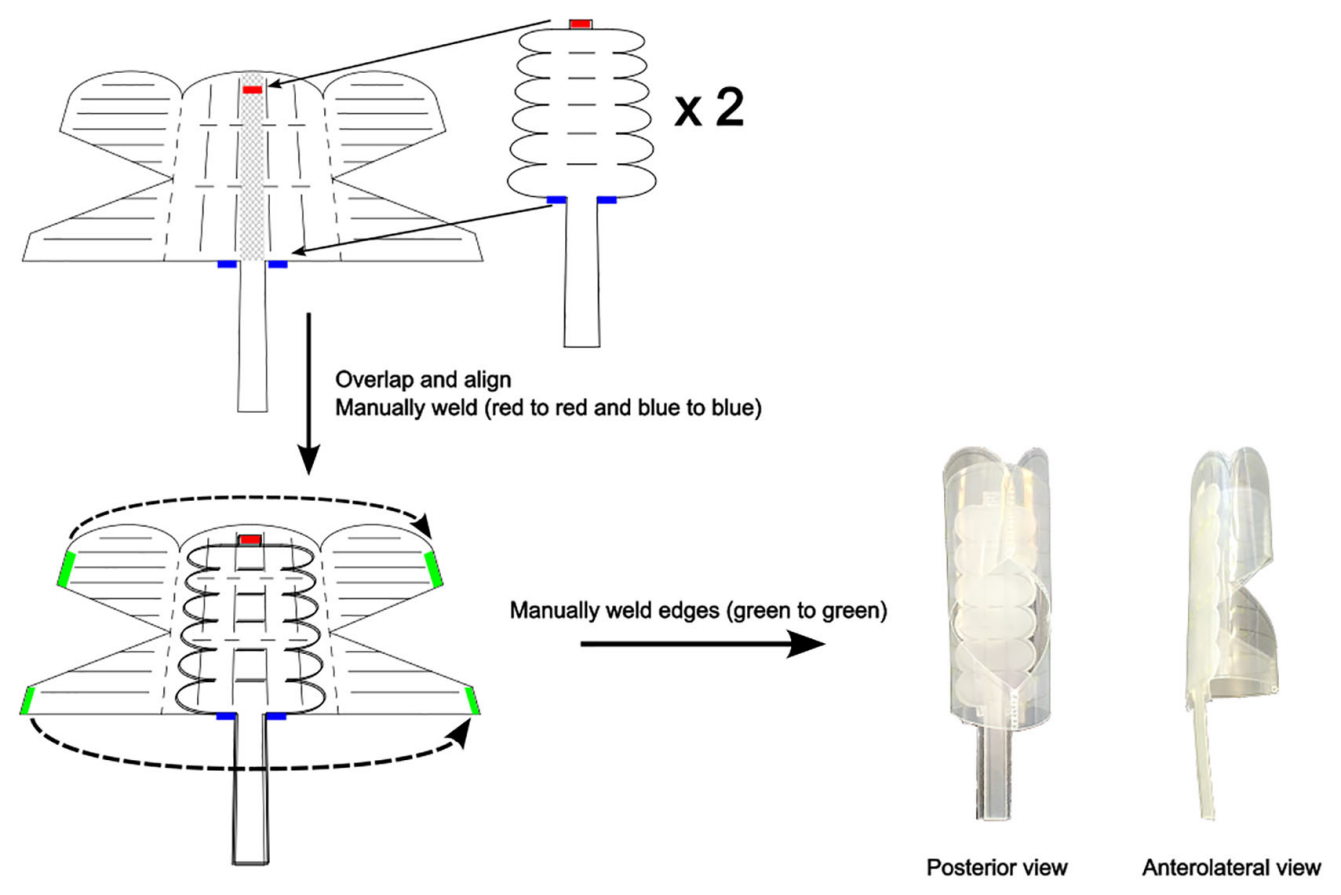
Fabrication of modified soft hydraulic endorectal actuator.

The endorectal actuator was fabricated using the same laser welding method as described in (31). The device has undergone five modifications. The extensor actuator was removed. The flexor actuator has elliptical ends instead of triangular ends, which reduces the risk of cutting the inner membrane of the main body. Two flexor actuators are used instead of one to increase the device’s ability to resist the opposite bending action. The main body consists of a three-sided dual-truncated prism instead of four sides. The device was found to undergo shearing on four sides, which a 3-sided device addresses. Finally, the tubing that hydraulically inflates the main body is pushed inside and all the way to the tip of the device. This allows us to push the device from the top of the device, similar to a urinary catheter. This pushing from the tip allowed the device to be inserted into the phantom. **Figure 3** shows the construction of the latest prototype.

### 2.5 Validation of device

The endorectal actuator was folded and inserted into the phantom by pushing the tubing into the rectum. The main body of the device burst between 15 ml and 16 ml of inflation. Therefore, once the device was inside the phantom, the main body was inflated to 14 ml to leave a safety buffer. Once inflated to this volume, the tubing was clipped shut to the main body to maintain a constant volume. The flexor actuators were then inflated to 4.4 ml, and the tubing was clipped shut. Once the endorectal actuator was inflated, its ability to reduce rectal motion in the phantom was evaluated.

#### 2.5.1 Dynamic compressions

The endorectal actuator was validated by simulating dynamic variations in the rectal volume and assessing the actuator’s ability to resist these rectal volume variations. As rectal motion and prostate motion are closely correlated, the rectal phantom was subjected to contractions by motor-controlled elastic ribbons that mimicked prostate intra-fraction motion (47, 48). The motors were programmed to induce rectal volume changes through compression, as outlined in **Table 2**. During each motion protocol, the rectal phantom was filmed from above using a smartphone camera placed 50 cm above the phantom platform and fixed to a tripod. This generated a beam-eye view of the AP plane. Videos were analyzed using Tracker software (49), with the rectal diameter, anterior rectal wall (ARW) position, and center of the proximal rectum tracked every 5th frame.

**Table 2:** Motion protocols simulated in the rectal phantom.

Motion type	Description	Contraction profile and timings.
Continuous drift	Slow, gradual change in rectal motion	Elastic ribbons contract slowly and continuously over 40 s
Transient excursion	Large motions, which rapidly resolve	Elastic ribbons rapidly contract down to 50% of the original diameter over 5 s, followed by 5 s relaxation back to baseline. Repeated once after 15 s
Persistent excursion	Constant, large displacement which does not resolve	Elastic ribbons rapidly contract down to 75% of original diameter over 5 s, maintaining this diameter for at least 30 s
High-frequency excursions	Repeated rapid motions over short intervals	Elastic ribbons rapidly contract down to 67% of original diameter, followed by immediate relaxation back to baseline. Repeated 3 times, 3 s apart.

#### 2.5.2 Reproducibility of rectal volume

The ability of the actuator to reproduce the rectal volume and anorectal angle during insertion and inflation was assessed. The actuator was inserted and inflated inside the phantom across the four levels of tone used prior to baseline to +3 revolutions around each motor shaft. The control was defined as the baseline tone + three revolutions. This assessed the ability of the device to reproducibly expand the rectum. At each tone level, rectal diameters were measured, and rectal volumes were calculated by assuming a perfect cylinder 80 mm in length. The difference in rectal volume from the control was used to estimate the expected prostate displacement associated with these volume changes. The extent of anterior prostate displacement is expected to be 0.06 mm per cm^3^ volume increase in rectal volume (50–52). Hence, the rectal volume difference was multiplied by 0.06 mm to calculate the expected prostate displacement at each tone level. Displacements were compared with and without the actuator inflated inside the phantom to assess the ability of the actuator to stabilize the phantom.

#### 2.5.3 Reproducibility of anorectal angle and ARW position

The proximal flange was moved on the optical platform to simulate changes in the anorectal angle and ARW position. Using the holes on the platform as a coordinate system, the flange was bolted at the following defined coordinates: the control position, as shown in **Figure 1**, was defined as (9,9); posterior rotation was at (10,9); and anterior rotation was at (9,10) and (8,10).

**Figure 4:**
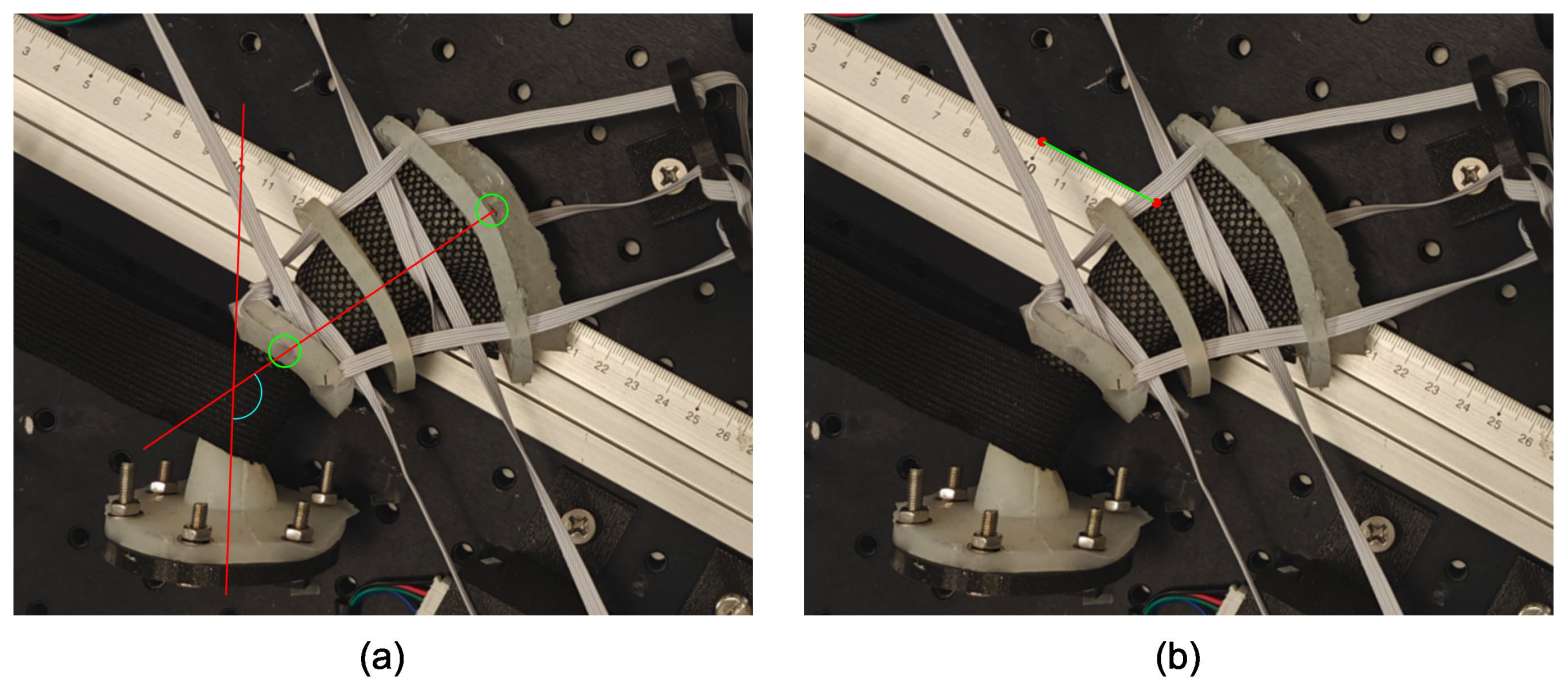
Measurements in the rectal phantom. **(A)** Measurement of anorectal angle and **(B)** position of the anterior rectal wall.

Images were captured from above using a tripod-fixed smartphone camera positioned 50 cm above the platform. A black pen was used to mark the top of the rectum. Using Inkscape software, a straight line denoting the rectal axis, was drawn on the image from this black mark to the center hole on the distal flange around the rectum (**Figure 4**). The anorectal angle was defined as the angle between the rectal axis and another line following the longitudinal axis of the anal canal. The ability of the device to reproduce this angle upon insertion was assessed by comparing these angles with and without the device. The ARW position reproducibility was also assessed. The position of the ARW was defined as the point at which the ARW of the phantom coincided with the top edge of the ruler in the image. Using a ruler as a reference, the position of the ARW in each case was compared with that in control to assess the actuator’s ability to “lock” in the rectum and consistently retract the ARW away from the target volume.

## 3 Results

### 3.1 Elastic ribbons

**Figure 5:**
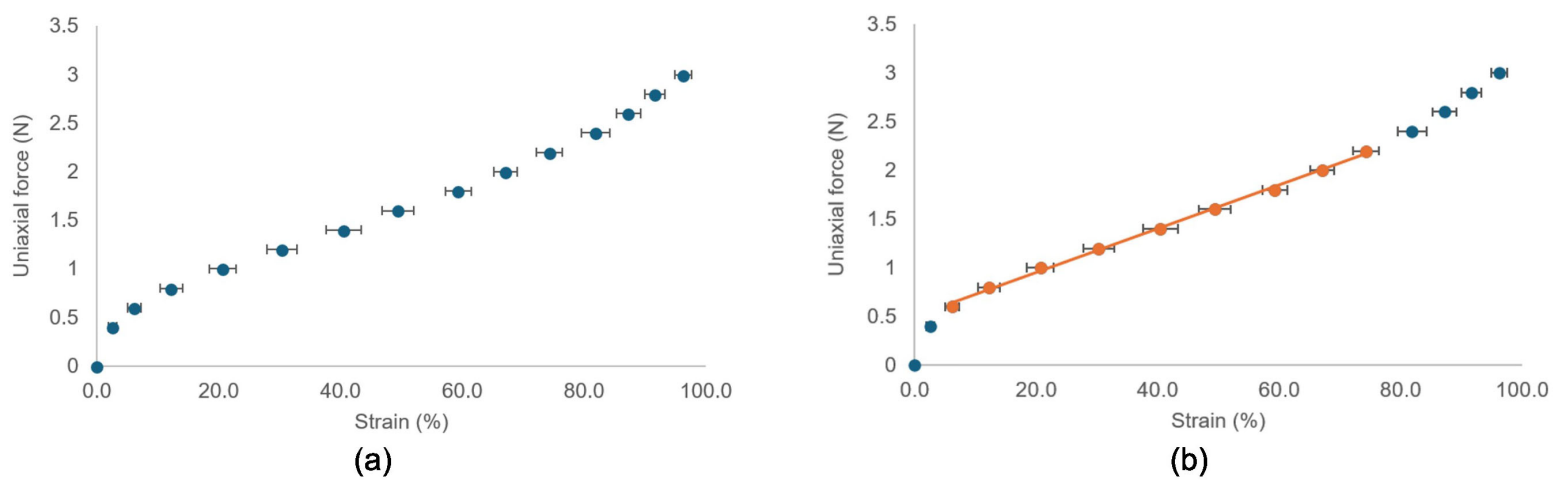
The force-strain curves of the elastic ribbons used in the rectal phantom. **(A)** Hyperelastic behavior is clear. **(B)** Linear part is highlighted in orange. Error bars are standard deviation from 20 trials.

The elastic ribbons consistently exhibited similar strains for a given force on the spring gauge. The averages for the 20 trials are shown in **Figure 5**, including the standard deviations denoted as error bars. **Figure 5** on the left shows the elastic ribbons exhibiting hyperelastic behavior. The stress–strain curve was steep at lower strains but then exhibits linear elastic behavior between 10% and 80% strain. Beyond 80% strain, the stress–strain curve exhibited a higher gradient. The linear region is highlighted on the right side of **Figure 5** and has an equation of F = 0.0224ϵ + 0.5031, where F is the force measured on the spring gauge required to stretch the ribbon to a strain, ϵ, expressed as a percentage. The strain was consistent, as indicated by the small standard deviation of the error bars. The used ribbons had slightly more strain (approximately 3% more in the linear region of the graph) than a “new” ribbon. This increase in strain did not occur if the ribbon was strained by no more than 70%. Beyond 70% strain, the elastic ribbons underwent plastic deformation, causing the initial length of the elastic ribbon to increase.

### 3.2 Barostat validations

The superior rectal diameter decreased with increased tone, but the inferior rectal diameter was mostly unchanged, as it was the minimum diameter of the barostat balloon. As mentioned, the first level of tone was established when the elastic ribbons were taut around the phantom. This was found when the elastic ribbons had 2.5, 2.5, 3.5, and 3.5 revolutions around the motor shafts labeled X, Y, Z, and E0, respectively, in **Figure 1**. **Table 3** shows the proximal and distal rectum’s radii, elastic ribbon strains, and corresponding forces associated with each level of induced tone. Ribbon lengths and strain in **Table 3** were calculated using the methods described in *section 2.3*. Both ribbons were within the linear strain region under 70% strain, allowing the force exerted by the ribbons to be determined using the gradient shown in **Figure 5**. In the remainder of this chapter, each tone level is referred to by its strain level. The rectal capacity and compliance at each volume range and the level of contraction of each elastic ribbon are shown in **Table 4**.

**Table 3:** Elastic ribbon strains and corresponding forces associated with each induced level of tone in the rectal phantom.

Revolutions around motor shafts.	Proximal rectum				Distal rectum			
	Radius (mm)	Ribbon length (mm)	Ribbon strain (%)	Ribbon force (N)	Radius (mm)	Ribbon length (mm)	Ribbon strain (%)	Ribbon force (N)
Just taut	26.25	597	-0.5	0.5	13.5	500	0	0.5
Taut + 1 revolution	24	646	7.6	0.7	13.25	574	15	0.8
Taut + 2 revolutions	18.5	687	14.4	0.8	12.75	658	32	1.2
Taut + 3 revolutions.	13	740	23.3	1.0	11.5	751	51	1.6

**Table 4:** Rectal compliance of the rectal phantom at different levels of muscular tone.

Elastic ribbon strain	Rectal diameter (mm)		Rectal capacity (ml)	Rectal compliance within volume range (ml/mmHg)		
	Proximal	Distal		40–60 ml	60–100 ml	100+ ml
No elastic	55	26	166	24.82	10.26	4.09
Baseline tone	52.5	27	140	4.19	3.11	1.92
Baseline + 1 rev	48	26.5	134	3.30	2.22	2.00
Baseline + 2 rev	37	25.5	136	2.92	2.47	2.17
Baseline + 3 rev	26	23	139	2.98	2.32	2.19

The rectal capacity was between 130 ml and 140 ml at all levels of tone. At baseline tone, the rectal compliance was 4.19 ml/mmHg between 40 ml and 60 ml. Rectal compliance decreased after one additional revolution around each motor shaft, but further additional revolutions did not have a significant effect on rectal compliance. Similarly, rectal compliance decreased as the volume of the rectum expanded. This was true for all tone levels. Over 100 ml, rectal compliance did not significantly differ between the different levels of tone as the volume of the balloon approached capacity. Without the elastic material around the rectal phantom, the rectal compliance was high at 24.82 and decreased sharply with increasing volume.

### 3.3 Dynamic compressions

**Figure 6:**
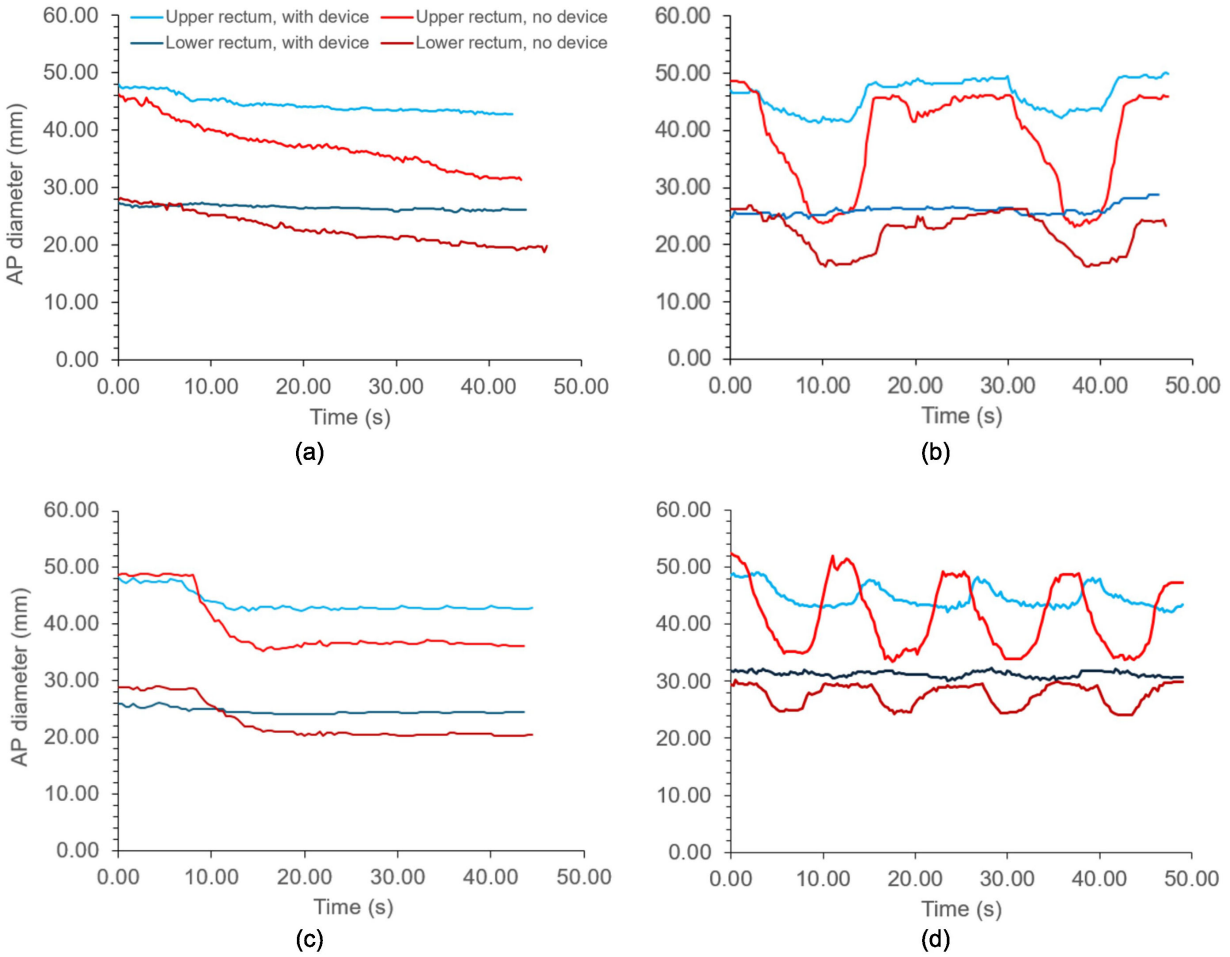
Diameters of the distal and proximal rectum with different motion protocols acting on the rectal phantom. **(A)** Continuous drift; **(B)** Transient excursion; **(C)** Persistent excursion; **(D)** High frequency excursion. Light blue: Proximal rectum, with device. Dark blue: Distal rectum, with device. Light red: Proximal rectum, no device. Dark red: Distal rectum, no device.

**Figure 7:**
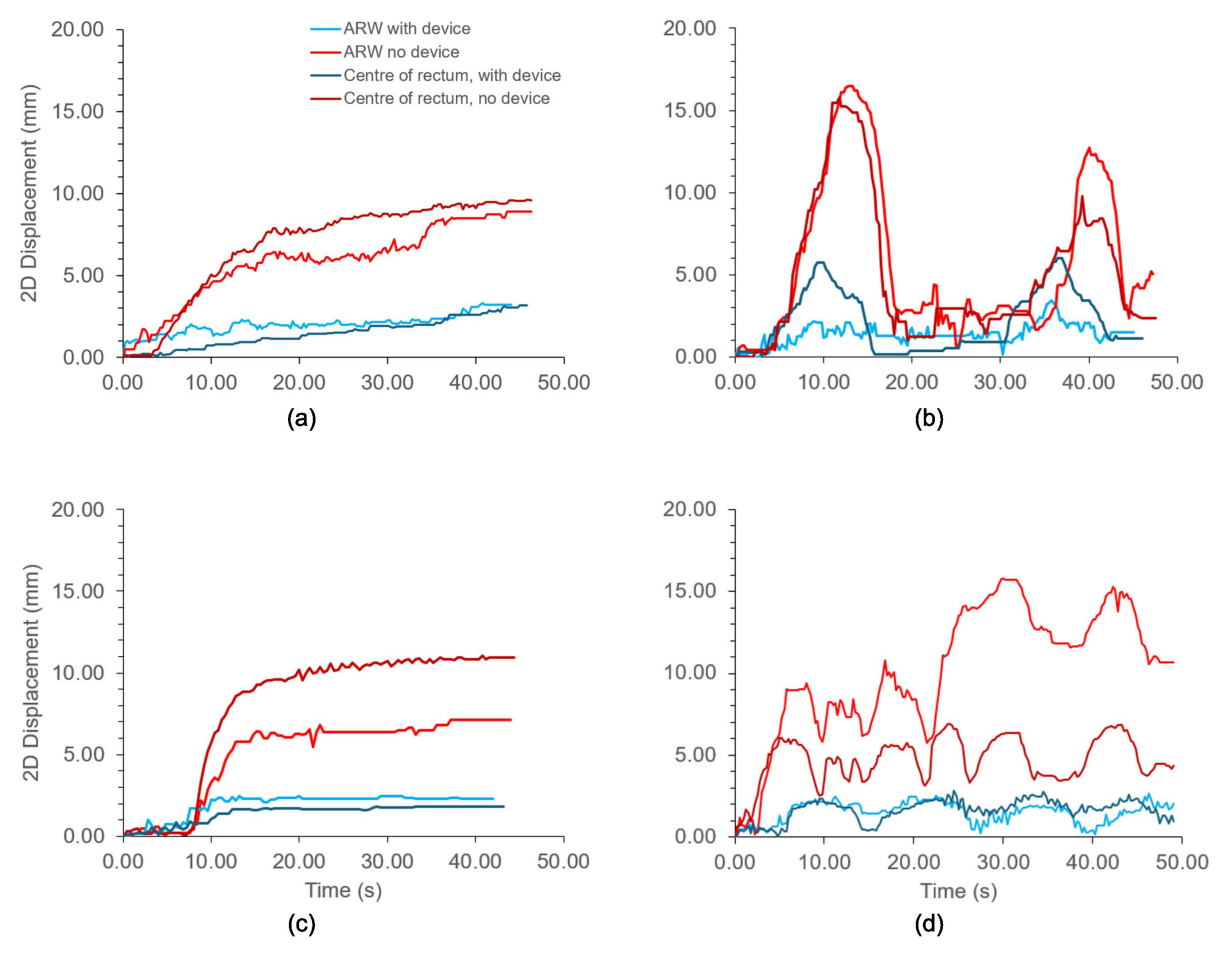
2-Dimensional displacements of the anterior rectal wall (ARW) and center of rectum with different motion protocols acting on the rectal phantom. **(A)** Continuous drift; **(B)** Transient excursion; **(C)** Persistent excursion; **(D)** High frequency excursion. Light blue: ARW with device. Dark blue: Center of rectum, with device. Light red: ARW, no device. Dark red: Center of rectum, no device.

**Figure 6** shows the changes in the anterior–posterior diameter over time in the proximal and distal rectum with and without the device for each motion protocol. **Figure 7** shows the same, but for two-dimensional displacements in the anterior–posterior direction for the anterior rectal wall and center of the rectum. In both graphs, divergence between the tracked positions can be observed between the rectums with and without the device. As shown in **Figure 6**, the diameter of the upper rectum consistently changed by less than 5 mm with the device, whereas without the device, the diameter changed by up to 25 mm, as shown in **Figure 6B**. Likewise, the ARW and center of rectum did not move more than 3 mm with the device inflated inside (**Figure 7**). Without the device, both the ARW and the center of the rectum consistently moved >5 mm, with the ARW displacing >15 mm at times (**Figures 7B, D**).

### 3.4 Reproducibility of volume and position

**Table 5** shows the diameters with and without the device at different extents of contraction by the elastic ribbons. Without the device, the rectum underwent a 132.3 cm^3^ change in volume, from 40.9 cm^3^ to 173.2 cm^3^. For this change in rectal volume, the prostate is expected to undergo an anterior translation of 7.94 mm. Using this device, the volume change between the control and lowest tone was 59.7 cm^3^. This change in volume would be expected to anteriorly translate the prostate by 3.58 mm. This is less than half the translation expected without the device for this extent of change in rectal volume.

**Table 5:** Outer diameter of the proximal rectum and calculated volume changes in the rectal phantom with and without the device at different extents of contraction.

Proximal rectum ribbon strain (%)	Measurement	Without device	With device
Control = 23.3%	Rectal diameter (mm)	25.5	42.5
	Volume (cm^3^)	40.9	113.5
	Est. prostate displacement relative to control (mm)	Control	Control
14.4%	Rectal diameter (mm)	37.0	43.0
	Volume (cm^3^)	86.0	116.2
	Est. prostate displacement relative to control (mm)	2.70	0.16
7.6%	Rectal diameter (mm)	48.0	48.0
	Volume (cm^3^)	144.8	144.8
	Est. prostate displacement relative to control (mm)	6.23	1.88
-0.5%	Rectal diameter (mm)	52.5	52.5
	Volume (cm^3^)	173.2	173.2
	Est. prostate displacement relative to control (mm)	7.94	3.5

Expected estimated prostate displacements as a result of volume changes from the control are included.

**Figure 8:**
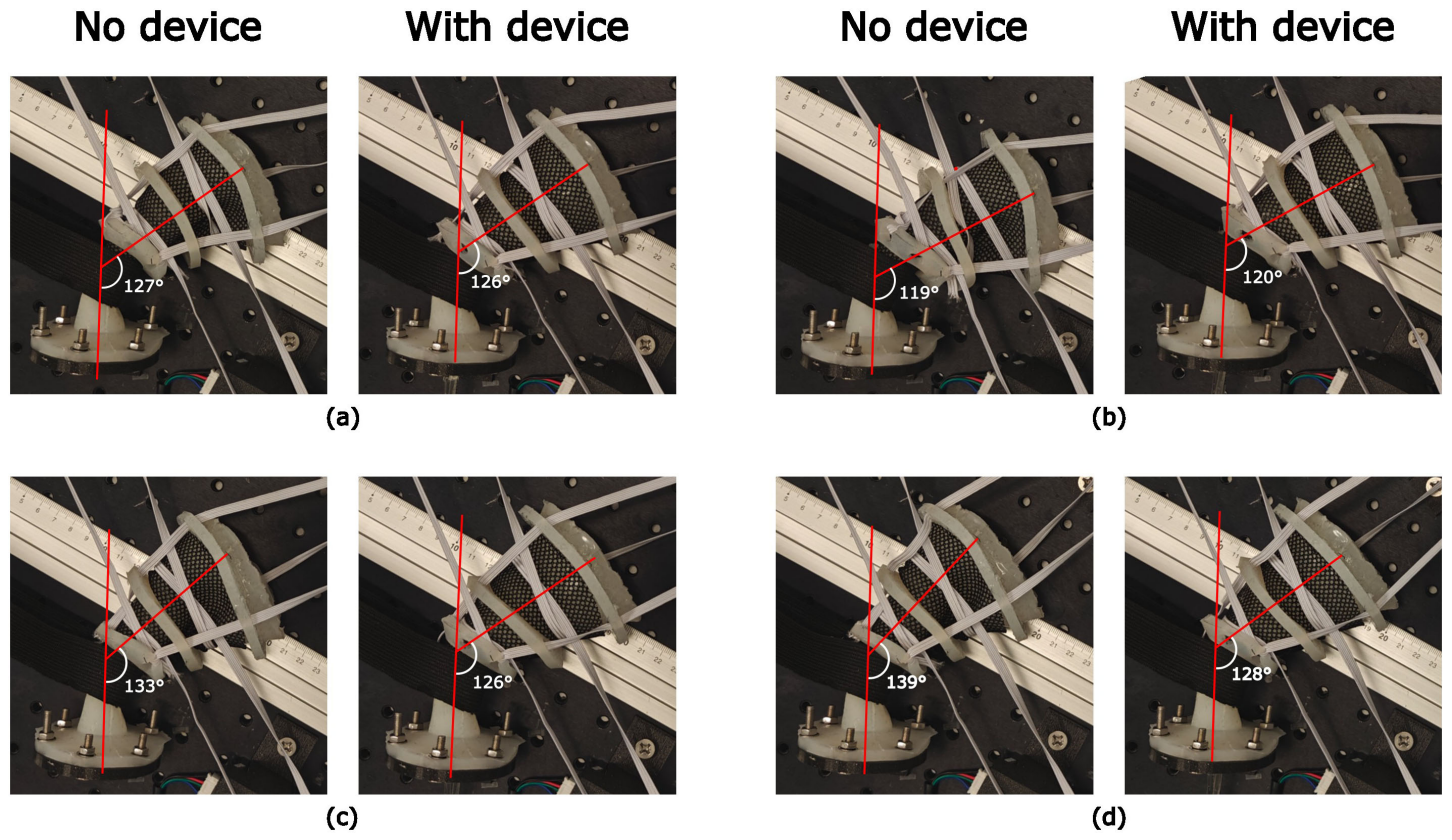
Anorectal angle positions with and without the device inside the rectal phantom when the proximal flange was moved to displace the rectal phantom. Device not visible as it is inside phantom. **(A)** Control position; **(B)** Posteriorly rotated phantom; **(C)** Anteriorly rotated phantom; **(D)** Further anterior rotation.

The ability of the device to reproduce the anorectal angle is shown in **Figure 8**. **Figures 8A, B** show that the anorectal angle of the phantom was similar with the device inside and without the device inside. **Figures 8C, D** show that the device reduced the angle of the phantom with significant anterior rotation. The anterior rotations increased the anorectal angle from 127° to 133° and 139°. In contrast, the angle did not change significantly from the control position when using the device. There was only a 2° increase in the anorectal angle between **Figures 8A, D**.

**Table 6** shows the anterior–posterior displacement in the ARW position, as defined in the *Methods* section, at different rectal positions. With anterior rotations, the ARW was displaced anteriorly by 7 mm and 13 mm compared to the control position. Compared to the control, when the device was inserted, the rectal phantom was translated by 3 mm and 4 mm.

**Table 6:** Anterior–posterior displacement of the anterior rectal wall (ARW) relative to control position in rectal phantom with and without the device.

Position of superior flange on platform (x, y)	ARW displacement (mm)	
	Without device	With device
Control (9, 9)	Control	Control
Posterior rotation (10, 9)	−8	−6
Anterior rotation (9, 10)	7	3
Further anterior rotation (8, 10)	13	4

Positive displacements are in the anterior direction.

## 4 Discussion

Our results demonstrate that the endorectal actuator effectively reduced motion in the validated rectal phantom, particularly the intra-fraction motion from simulated peristaltic forces. By minimizing variations in the rectal diameter (**Figure 6**), the device is expected to reduce prostate motion errors during radiotherapy fractions. Additionally, it limited anterior displacement of the ARW during each simulated fraction (**Figure 7**), thereby reducing the likelihood of the ARW entering the target volume. This is especially significant in the postsurgical setting, where the ARW delineates the target volume.

The phantom was validated using a barostat balloon, exhibiting rectal compliance in the lower end of the healthy range (3 ml/mmHg–15 ml/mmHg) (39). Although this suggests a higher resistance to distension than that of an average patient, it is unclear at what volume this compliance range applies. Indeed, our phantom exhibited two features of the human rectum: a decrease in compliance with increasing cross-sectional area or volume (32) and a rectal capacity of 134 ml–140 ml at 40 mmHg, which is within the normal range of 100 ml–250 ml (39). Despite the phantom’s higher resistance to distension, the actuator successfully expanded the rectum across various tone levels, as shown in **Table 5**. If real rectums are more compliant, the device should perform at least as well *in vivo*. By reproducibly expanding the rectum, the device reduces variations in rectal volume between fractions that would be caused by gas or peristaltic relaxation. **Figure 8** and **Table 6** also show that the device reproduces the anorectal angle and ARW position upon insertion. When the phantom was anteriorly rotated compared to the control position, the actuator “locked in” the anorectal angle at a consistent position. This consistency could reduce the inter-fraction motion within a patient and provide a consistent position across patients with differing anatomies. This reproducible positioning, particularly the reduction in anterior displacement of the ARW, is likely to prevent the rectum from moving into the target volume, potentially improving outcomes by reducing rectal toxicity.

Despite efforts to develop and validate biomechanically accurate rectal phantoms, several limitations remain. First, the phantom did not reproduce physiological temperatures (37°C), resulting in an approximate 15°C increase from the room temperature. Under constrained-volume conditions, our endorectal actuator when it is clamped shut after inflation, such a temperature increase would theoretically induce a pressure increase of 7 MPa–8 MPa. The thermal expansion was estimated using a linear thermoelastic relation. This pressure increase was more than an order of magnitude higher than the typical operating pressure of the endorectal actuator (0.155 MPa–0.175 MPa (31)). Such an increase in pressure can lead to device failure. Clinically, this necessitates adjustments to the inflation protocol: the actuator should be inflated very gradually to allow thermal equilibrium *in vivo*, or the water with which the device is inflated should be maintained at 37°C. Because the inflation of the endorectal actuator is pressure-limited, to reduce the risk of bursting, there is a temperature-dependent change in the achievable inflation volume. The bulk modulus of water is approximately 3% higher at 37°C than at 22°C (53), corresponding to a small change in the achievable inflation volume (<0.5 ml for a 14 ml inflation). While this change is unlikely to impact rectal stabilization, inflation protocols will require adjustments for human trials. Future burst-pressure and burst-volume investigations with water at 37°C are warranted to further validate device safety. The second limitation is that the phantom does not simulate anorectal humidity, which may affect the ease of insertion of the endorectal actuator compared to *in vivo* conditions. Although the actuator requires a soft silicone sleeve or external shell to cover the sharp plastic edges, the insertion methods used in this study are representative of the intended clinical process. Finally, we used a simple water-based lubricant to simulate rectal mucosa. This finding is consistent with those of previous colorectal phantom studies (54–57). While this approach does not replicate the complex rheological and adhesion properties of rectal mucus, which are important when studying microscopic behavior, such as particle transport and penetration (58), the primary objective of our study was to assess the endorectal actuator’s ability to stabilize the rectum following device insertion and inflation. Variations in rectal motion are primarily driven by peristalsis and gas production. For this mechanically focused goal, using a simple water-based lubricant was sufficient and does considerably affect our conclusions.

To our knowledge, this endorectal actuator is the first and continues to be the only deployable rectal stabilizer intended for use in prostate radiotherapy. This study is the first published study of its kind that assessed the rectal stabilizer in a rectal phantom *in vivo* simulation. Our findings support the potential of the device to reduce variations in rectal volume and, therefore, reduce target motion errors during prostate radiotherapy. Future studies will evaluate the effects of the device on radiotherapy and imaging. This evaluation includes whether it alters the dose distribution, scatter, or beam attenuation during radiotherapy. A preliminary unpublished study found that the current prototype of the endorectal actuator did not introduce artifacts or distortion on CBCT imaging. We will also focus on improving device usability. A soft silicone sleeve or external shell covers the plastic edges for improved safety and easier insertion. This will enable us to obtain ethical approval for human trials. Human trials will involve the assessment of the device’s safety and tolerability in a phase I trial and a subsequent phase II trial comparing this actuator to a control group and other devices, such as ProSpare™ or ERBs. A current limitation is the need for multiple syringes to operate the device, which prevents patient self-insertion. To address this, future versions should inflate the device with a single syringe, possibly by embedding automatic valves that control the direction of the flow.

## 5 Conclusion

The endorectal actuator demonstrated its ability to reduce variations in rectal motion in this phantom by stabilizing the rectal volume and position. These findings support the potential of the device to improve target accuracy and reduce ARW exposure in prostate radiotherapy. The device is translatable with improved usability for self-insertion by patients. Future clinical trials are required to validate these findings.

## Data Availability

The raw data supporting the conclusions of this article will be made available by the authors, without undue reservation.
